# Knowledge, attitudes, and practices of Texas hunters: a potentially high-risk population for exposure to the parasite that causes Chagas disease

**DOI:** 10.1186/s13071-015-0815-4

**Published:** 2015-04-01

**Authors:** Melissa N Garcia, Sarah K Murphy, Andrew Gross, Joel Wagner, Kristy O Murray

**Affiliations:** Baylor College of Medicine, National School of Tropical Medicine, 1102 Bates Avenue #550, Houston, Texas 77030 USA; University of Texas Health Science Center, School of Public Health, Houston, Texas 77030 USA; Lavaca County Wildlife Management Association, Hallettsville, Texas 77964 USA

**Keywords:** Chagas disease, Trypanosoma cruzi, Triatominae, Hunters, Disease transmission, Sylvatic transmission

## Abstract

**Background:**

Chagas disease is a parasitic infection transmitted to humans and mammals by the Triatominae insect. If untreated, Chagas disease can lead to heart failure and death. Previous publications highlighted the potential public health risk of disease transmission among hunters in the United States.

**Findings:**

We further investigated this population’s risk by administering a knowledge, attitudes, and practices questionnaire. Responses from hunters detailed the vector exposure and hunting practices unique to this population that might lead to their increased risk of disease transmission.

**Conclusions:**

Hunters should be aware of their potential risk for exposure to the insect that could be infected with the parasite that causes Chagas disease.

Chagas disease is a vector-borne disease caused by infection with the parasite *Trypanosoma cruzi* (*T. cruzi*). Humans most commonly acquire the infection from insect species in the Triatominae family, or rarely through alternate transmission sources (infected blood products or organs, congenital infection, and oral consumption of contaminated food products). Triatominae species are mostly nocturnal and feed on the blood of mammals. All five nyphmal instars and adult females take blood meals [1]. It is critical to understand which populations are at risk for infection considering up to 30% of people with Chagas disease will develop severe cardiac manifestations characterized by conduction abnormalities and progressive cardiomyopathy [2]. Without treatment, this heart condition can be fatal.

Domestic transmission occurs when the vector has adapted to living in human dwellings or nearby in animal enclosures [1]. Conversely, sylvatic transmission can result in human infection when humans interrupt the transmission cycle by entering the natural wildlife habitat of vectors and mammalian reservoirs [1]. Blood meal analysis of *T. cruzi* infected vectors from central Texas indicated high mammalian host diversity and a risk for disease transmission to humans [3]. Recent reports of locally acquired cases in the United States have highlighted the emergence of this disease in humans [4,5]. Despite these findings, very little research has been done to identify those at risk for disease transmission.

A study to assess transmission sources of infected blood donors in Houston, Texas reported one patient that regularly used a Triatominae infested hunting lease [5]. When three collected vectors from this hunting lease in Lavaca County, Texas, tested positive for *T. cruzi*, we began to investigate the possibility of hunters as a high-risk group for disease transmission. Based on our preliminary findings, we hypothesized that hunters could be a high-risk group for *T. cruzi* infection as a result of (1) increased opportunities of exposure to the vector, and (2) risk of blood-borne infection during the skinning process of potentially infected mammals [6].

To better assess this potential for increased risk, we developed a questionnaire to distribute at the 2014 annual meeting of the Lavaca County Wildlife Management Association (LCWMA). The mission of the LCWMA is to foster conservation of wildlife populations and their associated habitats, on member-owned or managed properties, and to encourage cooperation among neighbors to achieve common wildlife management goals. At the meeting, we distributed the questionnaire to determine the prevalence of hunting in this population, hunting practices potentially associated with disease transmission including vector exposure, overnight dwelling conditions, and types of animals hunted, as well as knowledge, attitudes, and practices of hunters regarding Chagas disease (*T. cruzi* infection). The questionnaire was approved by the Baylor College of Medicine institutional review board.

## Findings

Approximately 400 LCWMA members attended the 2014 meeting. Of the 207 adult participants who completed the survey, 189 (91%) reported being a hunter and were included in our subsequent analysis (Table 1). The hunters were primarily male (89%) with an average age of 55 years (range 19–82 years). They were mostly healthy, with only 20% reporting health conditions dominated by hypertension, diabetes, and non-specific heart conditions. The majority were born in Texas (92%) and all currently lived in southeast Texas. Twenty percent had lived, at some point in their lives, in another state with reported Triatominae species [1]. Thirty-eight percent reported a history of travel to an endemic Latin American country; however, the duration and reason for visit were unknown.

**Table 1:** **Potential high risk activities for** ***T. cruzi*** **infection**

	**Overall cohort**
***Vector exposure***	
Average duration of hunting (years, range)	40 (1–73)
Average hunting trip (days, range)	3 (1–30)
Ever seen vector	31% (53/172)
Ever bitten by vector	3% (6/180)
See woodrats in hunting areas	44% (77/177)
Use hunting stand	96% (181/189)
***Animals hunted*** ^***ǂ***^	
Game animals	93% (175/189)
Exotic animals	83% (156/189)
Non-game animals	66% (124/189)
Birds	50% (95/189)
Fur-bearing animals	38% (71/189)
***Hunting practices***	
Stay overnight while hunting	69% (130/189)
Sleeping structure normally infested with insects	18% (21/117)
Field dress animal	93% (175/189)
Never wear gloves when field dressing animal	68% (94/139)

^ǂ^Animal classifications defined by Texas Parks and Wildlife [9].

Vector exposure was assessed via lifetime hunting duration, direct contact with vector habitat (use of hunting stands and types), and regular sighting of vector and/or primary vector habitats (reservoir burrows). Participants reported hunting an average of 40 years (range 1–73 years) with a typical hunting trip lasting an average of 3 days (range 1–30 days). Most participants started hunting in youth (average age 16 years, range early childhood to 56 years). Box stands were the most common hunting structure used (83%), followed by tree stands (19%), and ground blinds (4%). Thirty-one percent of the hunters reported having seen the vector before. Six hunters reported being bitten by the vector, and only one of these six had been tested for Chagas disease. Forty-four percent of hunters reported seeing woodrats (*Neotoma* spp.), an important sylvatic reservoir, where they hunt [7]. Woodrats are insectivores whose burrows can be commonly infested with Triatominae species [7,8].

Overnight habitats during hunting were assessed, as the vector is a nocturnal feeder. Sixty-nine percent of hunters report staying an average of 2 nights per hunting trip. Thirty-four percent reported using a permanent housing structure, such as a camphouse, cabin, and/or lodge. Thirteen percent reported using a trailer. Tents were rarely used, only being reported by two participants. Of concern, eighteen percent of overnight hunters reported sleeping in an insect infested sleeping structure. Only permanent housing structures were reported to be infested.

Lastly, animals hunted and skinning practices were assessed to ascertain risk of blood borne transmission. Game animals, as defined by Texas Parks and Wildlife [9], were the most commonly hunted animals (93%), followed by exotic animals (83%), non-game animals (66%), birds (50%), and fur-bearing animals (38%). In the United States, there are 24 known competent mammalian hosts for *T. cruzi* infection [1]. Of these known wildlife hosts, ten are hunted year round in Texas. Out of concern for parasitemic blood-borne transmission during the skinning process, we evaluated the prevalence of these ten hunted mammalian hosts. Coyotes (*Canis latrans*) were the most commonly hunted (47%), followed by raccoons (*Procyon lotor*) (31%), nine-banded armadillos (*Dasypus novemcinctus*) (25%), bobcats (*Lynx rufus*) (24%), opossums (*Didelphis virginiana*) (21%), Mexican ground squirrels (*Spermophilus mexicanus*) (17%), skunks (*Conepatus* spp., *Mephitis* spp., *Spilogale* spp.)(16%), and foxes (*Canis* spp.)(6%). Badgers (*Taxidea taxus*) and ring-tailed cats (*Bassariscus astutus*) were infrequently hunted by our population (<3%). The majority (93%) of the hunters skinned deceased animals in the field. Glove use during the skinning process was uncommon. Sixty-eight percent of hunters never used gloves, 14% sometimes used gloves, and 18% always used gloves.

## Discussion

In Texas, autochthonous human cases of Chagas disease date back to 1955 [10,11]. Vector abundance, supportive ecologic niches, and readily available mammalian hosts throughout the state likely promote the establishment of a continuous sylvatic transmission cycle [1,3,12,13]. Previously reported locally acquired human cases have a high prevalence of outdoor activity and seeing wild reservoir animals on their property [4,5]. Hunters spend 4.6 times more hours outside than the average American, resulting in a longer risk of exposure to the vector [14,15]. Our population began hunting in their youth and continued the activity throughout the duration of one’s life. Participants spent an estimated average of 3,000 hours hunting, with most hunting occurring during overnight trips. With a third of the hunters having reported seeing the vector before and up to 82% of collected Triatominae vectors in Texas positive for the parasite that causes Chagas disease [3,16], we would argue that disease transmission is likely in this population.

In addition to direct vector exposure, hunters might have alternative exposure sources. Camphouses were used by a third of our population. If these overnight sleeping quarters are uncommonly used during non-hunting seasons, there could be an increased risk for Triatominae infestation. One might be at a higher risk for unsafe hunting and skinning practices resulting in hunting accidents and increased risk of exposure to potentially infectious blood. With two-thirds of our population not using gloves during skinning, and a high prevalence of hunting known *T. cruzi* mammalian reservoirs, blood-borne exposure could be an important risk factor for transmission in this population. Furthermore, 36% of our population reported travel to an endemic country. While we do not know the duration and reason for this travel, we cannot rule out this additional potential exposure source.

Another potentially important alternate exposure could be from hunting dogs. With 8.8% of shelter dogs infected with *T. cruzi* in Texas, the interplay between sylvatic, peri-domestic, and domestic settings should be further explored in relation to hunters [17]. Dogs are important in peri-domestic settings, particularly if they serve as a bloodmeal source for sylvatic vectors and subsequently as a potential reservoir for naïve vectors in a domestic setting. Dogs have been identified as bloodmeal sources in 75% of *T. cruzi* positive Triatomas in Texas and in 66.7% in the neighboring state of Louisiana [3,18]. Additionally, a high prevalence of rural owned hunting dogs in the neighboring state of Oklahoma were *T. cruzi* positive [19].

The primary purpose of this assessment was to understand the common practices of hunters and how they may relate to increased risk of acquiring *T. cruzi* infection. However, a major limitation of our study was not being able to test our hunters or their game for *T. cruzi* infection. Future studies should assess the seropositivity rate in this unique population as well as among the most commonly hunted game. To our knowledge, no studies have assessed white-tailed deer, the most hunted animal in our study, as a possible reservoir for infection. Additionally, there is a potential for selection bias as other hunter populations from different geographic regions and those whom use public hunting areas were not included in this assessment. These alternate populations should be evaluated for risk factor verification.

Based on our assessment, multiple practices might result in an increased transmission risk among hunters. These include extensive outdoor exposure in rural areas, skinning and field dressing potentially infectious game without gloves, and staying in potentially Triatominae-infested housing during overnight hunting trips. While a quarter of hunters reported knowledge of Chagas disease, there is still a need for disease education among this population to prevent future transmission. Forthcoming studies should aim to further investigate the relationship of hunting related behaviors and the risk for *T. cruzi* infection in the southern United States.
